# Pars plana vitrectomy for tractional and combined tractional rhegmatogenous retinal detachment in type 1 diabetes mellitus: outcomes, prognostic factors, and a proposed staging system

**DOI:** 10.1186/s40942-026-00835-0

**Published:** 2026-05-05

**Authors:** Mohammed S. Alshehri, Adhwa Alsadoon, Alanaud Albazei, Turki Algethami, Marco Mura, Sulaiman M. Alsulaiman, Faisal S. Alqahtani

**Affiliations:** Vitreoretinal Division, King Khaled Eye Specialist Hospital, Riyadh, Saudi Arabia

**Keywords:** Pars plana vitrectomy, Type 1 diabetes mellitus, Tractional retinal detachment, Tamponade, Ellipsoid zone, External limiting membrane

## Abstract

**Background:**

Pars plana vitrectomy (PPV) in young type 1 diabetes mellitus (T1DM) patients presents greater surgical challenges than in type 2 (T2DM) cases. Despite advances in vitreoretinal surgery, there is no universally accepted classification system for tractional retinal detachment (TRD), and limited data exist on the comparative effectiveness of endotamponade agents and the prognostic significance of outer retinal biomarkers in T1DM cohorts. This study evaluates PPV outcomes for TRD and combined tractional rhegmatogenous retinal detachment (TRRD) in T1DM, introduces a proposed staging system for TRD, assesses the impact of tamponade selection on outcomes, and investigates the role of outer retinal structural biomarkers in predicting visual success.

**Methods:**

A retrospective cohort study was conducted at King Khaled Eye Specialist Hospital, Riyadh, Saudi Arabia, on T1DM patients treated with PPV for TRD and combined TRRD between June 2014 and December 2022. A novel TRD staging system was proposed based on the extent of the tractional membrane, posterior hyaloid attachment status, and the presence of retinal breaks, classifying cases into simple TRD (stages 1, 2, 3a), complex TRD (stages 3b, 4, 5), and combined TRRD (acute and chronic). Anatomical success was defined as retinal reattachment, and visual success was defined as improvement in visual acuity of more than two Snellen lines. Postoperative optical coherence tomography (OCT) at 1 year assessed external limiting membrane (ELM) and ellipsoid zone (EZ) integrity.

**Results:**

Among 339 eyes (255 patients), complex TRD was the most common presentation (41.6%). Iatrogenic retinal breaks occurred in 39.2%, and tamponade was used in 74.0% of cases. Anatomical success was achieved in 73.2% after a single surgery and 91.2% overall. Visual success was achieved in 55.2% of eyes, with vision improvement observed in 69.2%. Eyes without tamponade achieved the highest visual success rate (80.8%), followed by gas tamponade (84.3% for C3F8/SF6) compared to silicone oil (51.9%) (*p* < 0.001). Combined TRRD had significantly lower visual success compared to complex TRD (47.3% vs. 64.5%, *p* = 0.018). Multivariate analysis identified preoperative LogMAR visual acuity (RR 9.88, 95% CI: 4.68–20.83, *p* < 0.001), intact ellipsoid zone status (RR 20.4, 95% CI: 3.8–109.41, *p* < 0.001), combined TRRD stage (RR 0.08, 95% CI: 0.01–0.5, *p* = 0.007), and absence of subretinal fluid (RR 3.49, 95% CI: 1.12–10.87, *p* = 0.031) as significant independent predictors of visual success.

**Conclusion:**

PPV for TRD in T1DM achieves excellent overall anatomical outcomes. The proposed TRD staging system demonstrated prognostic value, with combined TRRD carrying a worse visual prognosis. Preoperative visual acuity, TRD stage, outer retinal integrity (ELM/EZ) on postoperative OCT, and tamponade type were significant determinants of functional outcomes. The association of silicone oil tamponade with poorer visual outcomes likely reflects indication bias, as silicone oil was preferentially used in more complex cases. The proposed staging system is hypothesis-generating and requires prospective multicenter validation.

## Introduction

Diabetic retinopathy (DR) is one of the leading causes of blindness worldwide [1]. In 2021, an estimated 529 million people globally had diabetes, with a prevalence of 6.1% across all ages [2]. The prevalence of diabetes mellitus increased by 90.5% from 1990 to 2021, rising from 3.2% to 6.1%. Looking ahead to 2050, the number of people with diabetes is projected to rise by 59.7%, reaching 1.31 billion, primarily driven by T2DM [2]. A systematic review found that over a third (34.6%) of individuals with diabetes have DR, with a significant proportion (10.2%) experiencing vision-threatening DR (VTDR) [3]. 

According to the Early Treatment Diabetic Retinopathy Study (ETDRS), 5% of patients with DR required vitrectomy even after receiving full scatter pan-retinal photocoagulation (PRP) [4]. Most of these patients were T1DM, with over half needing the procedure. Additionally, the Diabetic Retinopathy Vitrectomy Study (DRVS) found that early vitrectomy offered more significant long-term benefits for T1DM patients experiencing vitreous hemorrhage [5]. 

Pars plana vitrectomy (PPV) poses more significant challenges in young patients with T1DM than those with T2DM. Huang et al. reported a recurrence rate of retinal detachment following PPV of 13.2% in T1DM patients, with an anatomical success rate of 91.2% [6]. Similarly, another study demonstrated poorer functional and anatomical outcomes in younger patients with advanced PDR compared to older patients [7]. Kazmierczak et al. evaluated the outcomes of PPV in T1DM and T2DM patients [8]. While both groups experienced significant improvement in mean postoperative best-corrected visual acuity (BCVA), the improvement was statistically more significant in the T2DM group.

Tractional retinal detachment encompasses a broad spectrum of clinical presentations, ranging from focal traction at the disc or arcades to diffuse fibrovascular proliferation with complete posterior hyaloid attachment. Several classification systems have been proposed to categorize TRD [9–13]. Kroll et al. proposed a classification system for proliferative diabetic vitreoretinopathy (PDVR) with three stages based on the extent of retinal involvement [8]. However, existing classification systems were developed in an era of different surgical techniques and may not adequately address the nuances of contemporary minimally invasive vitreoretinal surgery (MIVS) PPV, particularly regarding hyaloid status, chronicity, and the distinction between tractional and combined rhegmatogenous components. To date, there is no universally accepted or validated TRD staging system.

Furthermore, data on the comparative effectiveness of intraocular tamponade agents in T1DM specifically remain limited. The integrity of outer retinal structures, particularly the external limiting membrane (ELM) and the ellipsoid zone (EZ), is emerging as an important predictor of visual outcomes following vitreoretinal surgery. However, there is insufficient understanding of these biomarkers in large TRD cohorts within T1DM.

This study aimed to: (1) evaluate the outcomes of PPV for TRD and combined TRRD in T1DM patients, (2) introduce a proposed staging system for TRD based on the extent of tractional membranes, posterior hyaloid attachment, and presence of retinal breaks, (3) assess the impact of different endotamponade agents on surgical outcomes, and (4) investigate the prognostic value of outer retinal structural biomarkers (ELM/EZ integrity) on postoperative OCT. We hypothesized that TRD complexity, tamponade choice, and the integrity of ELM/EZ would be independently associated with visual outcomes following PPV in T1DM.

## Methods

This study received approval from the institutional review board of King Khaled Eye Specialist Hospital and adhered to the tenets of the Declaration of Helsinki. A single-institution retrospective cohort study was conducted on patients with T1DM who underwent PPV for TRD and combined tractional rhegmatogenous retinal detachment (TRRD) between June 2014 and December 2022 at King Khaled Eye Specialist Hospital, Riyadh, Saudi Arabia. It should be noted that surgical techniques, instrumentation, and clinical protocols may have evolved over the 8-year study period, which is acknowledged as a potential source of variability.

Patients with T2DM and those who underwent vitrectomy for other reasons, such as vitreous hemorrhage (VH), were excluded. Additionally, patients with concurrent ocular morbidities (retinal vascular occlusion, optic neuropathy, glaucoma), those with less than six months of follow-up after their last vitreoretinal intervention, and patients with silicone oil in situ were excluded. Eyes with pre-existing silicone oil in situ were excluded because these represent a distinct clinical scenario of re-detachment or failure of a prior intervention, which would introduce significant heterogeneity and confounding to the analysis of primary PPV outcomes.

Preoperative data collected included age, gender, duration of diabetes, concurrent diabetic nephropathy, and prior interventions for diabetic retinopathy. Best corrected visual acuity (BCVA), clinical examination findings, and preoperative optical coherence tomography (OCT) findings were documented. Preoperative ultrawide field fundus photos were used to determine the stage of TRD and the number of involved quadrants. B-scan ultrasonography was used to evaluate the status of the posterior hyaloid.

**Fig. 1:**
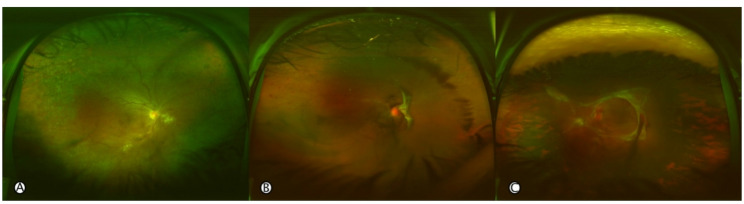
Color fundus photographs illustrating simple tractional retinal detachment (TRD) stages according to the proposed staging system. **A**: Stage 2: Focal TRD inferonasal to the optic disc without macular involvement, with peripheral laser marks (right eye). This stage represents localized traction without broad fibrovascular adhesion. **B**: Stage 3 A: Broad fibrovascular adhesion at the optic disc with dispersed vitreous hemorrhage, microaneurysms, and intraretinal hemorrhages temporal to the macula (right eye). This stage demonstrates broad arcade/peripheral traction without direct macular involvement. **C**: Stage 3B: Broad fibrovascular adhesion involving the macula, optic disc, and both arcades with dispersed vitreous hemorrhage (left eye). Stage 3B marks the transition from simple to complex TRD, with macular involvement

**Fig. 2:**
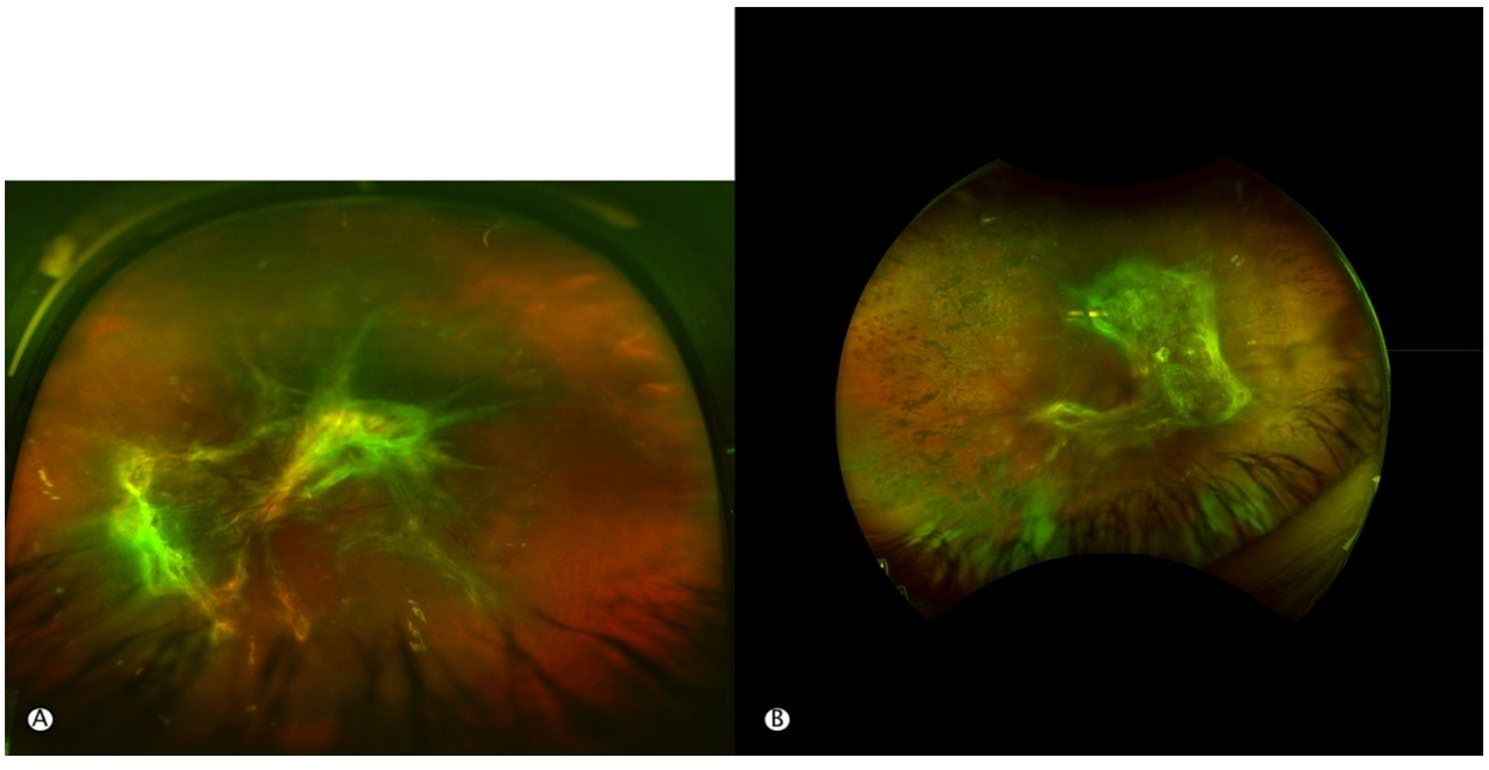
Color fundus photographs illustrating complex tractional retinal detachment (TRD) stages according to the proposed staging system. **A**: Stage 4: Broad fibrovascular proliferation and TRD involving the macula, optic disc, and arcades extending to the peripheral retina, with partial posterior vitreous detachment confirmed by B-scan (left eye). Stage 4 is distinguished from stage 5 by the presence of partial PVD. **B**: Stage 5: Broad fibrovascular proliferation and TRD involving the macula, optic disc, and arcades extending to the peripheral retina, with old dehemoglobinized blood inferiorly and laser marks; no posterior vitreous detachment by B-scan (right eye). Stage 5 represents the most complex form, with complete hyaloid attachment increasing surgical difficulty

**Fig. 3:**
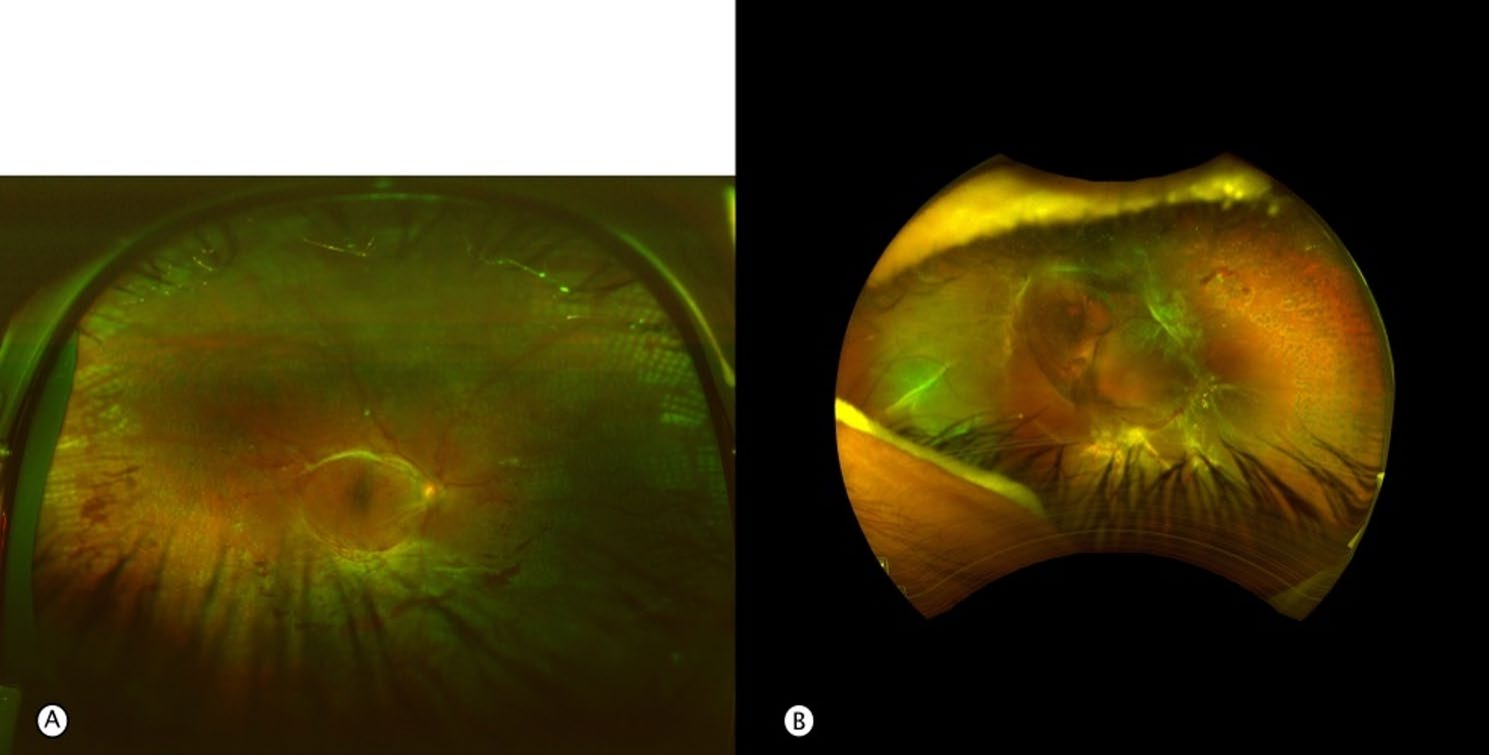
Color fundus photographs illustrating combined tractional rhegmatogenous retinal detachment (TRRD) according to the proposed staging system. **A**: Acute combined TRRD: Inferior TRD with corrugated retina, fibrovascular membrane over superior arcade, preretinal hemorrhage, and fresh laser marks (right eye). Acute TRRD is defined as retinal break with detachment of less than one week duration. (N.B. break is not clearly visible in the color fundus photo; however, it was documented in the preoperative clinical examination.) **B**: Chronic combined TRRD: TRD involving the macula and both arcades, associated with vitreous hemorrhage, detached retina nasally with subretinal band, and temporal laser marks (left eye). Chronic TRRD is defined as retinal break with detachment persisting for more than one week, and carries a worse visual prognosis

The stage of TRD was assessed based on the extent of the tractional membrane, attachment of the posterior hyaloid, and the presence of retinal breaks following a proposed staging system (Table 1). This staging system is proposed as a hypothesis-generating framework for standardizing the recognition of different TRD presentations and their surgical complexity. It aims to improve diagnostic precision by incorporating surgical anatomical considerations specific to contemporary MIVS PPV. The system has not yet been validated in prospective studies, and inter-observer agreement data are not available. Acute combined tractional rhegmatogenous retinal detachment (TRRD) was defined as detachment lasting less than one week, while symptoms persisting for more than a week were considered chronic. Stages 1, 2, and 3a were classified as simple TRD, while stages 3b, 4, and 5 were classified as complex TRD. Surgical indications for simple TRD cases included progression of traction threatening the macula, documented progression of extramacular TRD on serial examinations, associated non-clearing vitreous hemorrhage causing significant visual impairment, and tractional macular edema refractory to medical therapy. Although many simple TRD cases may appear to have a relatively limited area of detachment, surgical intervention was warranted when there was evidence of progressive traction or when the clinical presentation posed a risk to central visual function. (Figures 1, 2, and 3)

**Table 1:** Preoperative staging system of tractional retinal detachment associated with diabetes mellitus

Stage	Definition
1	Vitreoretinal interface pathology (epiretinal membrane, vitreomacular adhesion, vitreomacular traction)
2	Focal TRD over disc, or arcade or periphery
3a	Broad fibrous membrane traction at arcade or periphery
3b	Broad fibrous membrane traction at macula with adhesion at disc, and or arcade
4	Stage 3b + extending to periphery with partial PVD
5	Broad fibrous membrane traction at disc, macula, and arcade extending to periphery without PVD
Acute CTRRD	TRD with retinal break for less than 1 week
Chronic CTRRD	TRD with retinal break for more than 1 week

Broad Adhesion is defined as more than two-optic disc diameter. TRD: tractional retinal detachment, PVD: posterior vitreous detachment, TRRD: combined tractional rhegmatogenous retinal detachment

Surgical procedures were performed using the Constellation Vision System (Alcon Laboratories, Fort Worth, TX, USA) with predominantly 23-gauge and 25-gauge instrumentation. Combined phaco-vitrectomy was performed when visually significant cataract was present at the time of surgery or when adequate surgical visualization required lens removal. Intravitreal bevacizumab (1.25 mg/0.05 mL) was administered preoperatively, with timing categorized as 3 days or less and more than 3 days before surgery. Tamponade selection (air, gas, or silicone oil) was at the discretion of the operating surgeon based on intraoperative findings and clinical judgment; no standardized protocol for tamponade selection was used.

Data on the timing and technique of surgical intervention were collected, including unimanual or bimanual dissection technique, intraoperative hemorrhage, intraoperative breaks, and type of tamponade used. Postoperative data included BCVA, IOP, anterior and posterior segment examination findings, and OCT findings. Data were recorded at the first postoperative visit (within one month of surgery), at 6 months, and at 1 year. The postoperative OCT results presented in this study are from the 1-year visit evaluation. The minimum follow-up of 6 months was chosen to maximize case inclusion while allowing adequate time for assessment of retinal reattachment stability. The 1-year OCT data represent a subset of patients who reached this time point. OCT assessments of ELM and EZ status were performed by two independent graders; however, the graders were not blinded to clinical data given the retrospective nature of the study.

In patients with bilateral involvement, each eye was treated independently based on its clinical presentation and both eyes were included in the analysis. The potential statistical implications of correlated data from bilateral eyes are acknowledged in the Limitations section.

We defined success based on three surgical outcomes: anatomical success after a single vitreoretinal intervention, overall anatomical success, and visual success, defined as an improvement in visual acuity of more than two lines on the Snellen chart.

### Statistical analysis

Data were collected, stored, and managed in a spreadsheet using Microsoft Excel 2010^®^ software. Data were coded and analyzed using SPSS^®^ version 21.0 (IBM Inc., Chicago, Illinois, USA). Descriptive analysis was performed, reporting proportions as numbers and percentages and continuous variables as mean ± standard deviation (SD) and range, following normality tests (Shapiro-Wilk test and Q-Q plots) that indicated normally distributed data. Consequently, one-way ANOVA (for three groups) and independent t-test (for two groups) were used to test differences between the means, while the Chi-squared test compared proportions. Any output with a p-value below 0.05 was considered statistically significant.

Given the exploratory nature of many of our analyses and the risk of inflating Type II errors, Bonferroni correction for multiple comparisons was not applied. However, findings from multiple comparisons should be interpreted with appropriate caution. The study included 84 patients with bilateral involvement (168 eyes); the potential clustering effect of bilateral eyes from the same patient is acknowledged as a limitation, and future studies should consider mixed-effects models to account for this correlation. Propensity score analysis for tamponade selection was not performed due to the complexity of surgical decision-making based on intraoperative findings not fully captured in the retrospective dataset; this is recommended for future prospective investigations.

## Results

A total of 339 eyes from 255 patients were included. Patient demographics and clinical characteristics are presented in Table 2. The majority of eyes presented with Snellen BCVA worse than 20/100 (281 eyes, 88.9%). Table 3 presents the evaluation of various factors in relation to the presenting BCVA.

**Table 2:** Demographics and clinical characteristics at presentation

Characteristics	*n* (%)
Age at presentation in years, mean ± SD [Range]	30.0 ± 6.6 [15.0-55.2]
Gender, n (%) (*n* = 255 patients)	
Male	136 (53.3)
Female	119 (46.7)
TRD side, n (%)	
Unilateral	171 (67.1)
Bilateral	84 (32.9)
Concurrent renal disease, n (%)	46 (18.0)
Duration of TRD in months, mean ± SD [Range]	4.2 ± 3.7 [1 day – 12 months]
Pre Op VA, n (%) (*n* = 316 eyes)	
NLP	0 (0.0)
LP	9 (2.8)
HM	81 (25.6)
CF	16 (5.1)
2/200-4/200	40 (12.7)
20/400 − 20/200	123 (38.9)
20/100	12 (3.8)
Better than 20/100	35 (11.1)
Pre Op VA LogMAR, mean ± SD [Range]	1.56 ± 0.69 [0.00–3.00] 20/710 [20/20-LP]

TRD: tractional retinal detachment, NLP: no light perception, LP: light perception, HM: hand motion, CF: counting finger, SD: standard deviation

**Table 3:** Factors associated with good Preop Visual acuity

Variable	Preop Visual acuity		*P* value
	> 20/100 (*n* = 35 eyes) *n* (%)	≤ 20/100 (*n* = 281 eyes) *n* (%)	
Macula status			
On	19 (26.0)	54 (74.0)	< 0.001*
Off	9 (4.9)	173 (95.1)	< 0.001*
Threaten the fovea	5 (16.1)	26 (83.9)	< 0.001*
Other – no view, ND and just other	2 (6.7)	28 (93.3)	< 0.001*
Stage of TRD			
1	0 (0.0)	5 (100)	< 0.001*
2	3 (37.5)	5 (62.5)	< 0.001*
3a	4 (17.4)	19 (82.6)	< 0.001*
3b	9 (20.0)	36 (80.0)	< 0.001*
4	2 (2.9)	66 (97.1)	< 0.001*
5	3 (16.7)	15 (83.3)	< 0.001*
Acute CTRRD	9 (45.0)	11 (55.0)	0.264
Chronic CTRRD	3 (4.7)	61 (95.3)	< 0.001*
Others	2 (3.1)	62 (96.9)	< 0.001*
Simple TRD (Stage 1 + 2+ 3a)	7 (19.4)	29 (80.6)	0.160
Complex TRD (Stage 3b + 4 + 5)	14 (10.7)	117 (89.3)	
Complex TRD	14 (10.7)	117 (89.3)	0.449
Combined TRRD (acute + chronic)	12 (14.1)	73 (85.9)	
Presence of vitreous hemorrhage			
Yes	16 (8.6)	170 (91.4)	0.094
No	19 (14.6)	111 (85.4)	

CTRRD: combined tractional rhegmatogenous retinal detachment, TRD: tractional retinal detachment

Preoperative evaluation details are presented in Table 4. Macula-off TRD was found in 199 eyes (58.7%), while 77 eyes (22.7%) had macula-on TRD. Few eyes had concurrent diabetic macular edema (DME) (*n* = 18, 7.1%). The most common presentation was complex TRD, affecting 141 eyes (41.6%). Combined TRRD was the second most common presentation, seen in 91 eyes (26.9%). Only 38 eyes (11.3%) had simple TRD at presentation. Stage evaluation of TRD was not possible for 69 eyes (20.7%) due to the lack of fundus photographs at presentation or an obstructed view of the fundus without ultrasonography.

**Table 4:** Preoperative evaluation

Characteristics	*n* (%)
Macula status (*n* = 339 eyes)	
On	77 (22.7)
Off	199 (58.7)
Threaten the fovea	31 (9.1)
Other – no view, ND and just other	32 (9.4)
DME (*n* = 255 patients)	18 (7.1)
Hyaloid status (122 eyes)	
Attached	30 (24.6)
Detached	92 (75.4)
Quadrants affected (*n* = 219 eyes)	
1 (No. of eyes and %)	28 (12.8)
2 (No. of eyes and %)	67 (30.6)
3 (No. of eyes and %)	35 (16.0)
4 (No. of eyes and %)	89 (40.6)
Stage of TRD (*n* = 339 eyes)	
1	5 (1.5)
2	9 (2.7)
3a	24 (7.1)
3b	49 (14.5)
4	73 (21.5)
5	19 (5.6)
Acute CTRRD	22 (6.5)
Chronic CTRRD	69 (20.4)
Others	69 (20.4)
Patient who received PRP (No. of eyes and %)	253 (74.6)
Patient who received injection (No. of eyes and %)	56 (16.5)
Time from presentation to surgery (days), mean ± SD [Range]	153.4 ± 221.6 [same day-1121 days]

ND: not documented, DME: diabetic macular edema, CTRRD: combined tractional rhegmatogenous retinal detachment, TRD: tractional retinal detachment, PRP: pan-retinal photocoagulation

Prior to the presentation, 253 eyes (74.6%) received pan-retinal photocoagulation (PRP), while only 56 eyes (16.5%) received an anti-vascular endothelial growth factor (VEGF) injection. The mean time between presentation and surgery ranged from the same day to 1121 days (153.4 ± 221.6 days).

Table 5 summarizes the intraoperative findings of the study. The bimanual membrane dissection technique was used in 271 eyes (85.5%). The bimanual technique was the most commonly used approach, regardless of the stage of TRD. A total of 23 eyes (60.5%) with simple TRD were managed using the bimanual technique, while 116 eyes (82.3%) with complex TRD also utilized this method. Additionally, 93 eyes (84.5%) with combined TRDD were treated with the bimanual technique.

**Table 5:** Intraoperative statistics

	*n* (%)
Bimanual technique (No. of eyes and %)	271 (85.5)
Unimanual technique (No. of eyes and %)	46 (14.5)
Intraoperative hemorrhage (No. of eyes and %)	34 (10.0)
Intraoperative break (No. of eyes and %)	133 (39.2)
Iatrogenic	122 (36.0)
Intentional	11 (3.2)
Retinotomy	9
Retinectomy	2
No. and % of eyes who received tamponade	251 (74.0)
Type of tamponade (*n* = 251 eyes)	
C3F8	34 (13.5)
SF6	23 (9.2)
SO 1300	128 (51)
SO 5000	51 (20.3)
Heavy SO	2 (0.8)
Other	13 (5.2)
AIR	8
Not mentioned	5
No. and % of eyes with cataract surgery	51 (15.0)

C3F8: perfluoropropane, SF6: sulfur hexafluoride, SO: silicone oil

Tamponade was used in 251 eyes (74.0%). Regular silicone oil (SO) was the most frequently used tamponade agent (179 eyes, 71.2%), followed by perfluoro propane (C3F8) (34 eyes, 13.5%) and sulfur hexafluoride (SF6) (23 eyes, 9.2%). Silicone oil was the most commonly used tamponade in both complex TRD and combined TRRD, accounting for 65 eyes (46.1%) and 88 eyes (80%), respectively. Additionally, no tamponade was used in 21 eyes (55%) with simple TRD and in 45 eyes (31.9%) with complex TRD. Table 6 details all postoperative complications encountered in the study.

**Table 6:** Post op complications

	*n* (%)
Post op complications	
None	62 (18.3)
Cataract	187 (55.2)
High IOP	44 (13.0)
SOAG	17 (5.0)
SCAG	7 (2.1)
Failure of reattachment	7 (2.1)
Post Op VH	28 (8.3)
SO emulsification	25 (7.4)
Recurrent RD	39 (11.5)
PVR	24 (7.1)
ERM	38 (11.2)
Residual SRF	32 (9.4)
Other	22 (6.5)
VH	2
CTRRD	1
TRD	3
SYMPATHETIC OPHTHALMIA	2
RRD	1
Recurrent detachment + SO emulsification + PVR+ Cataract	1
Persistent CED	1
NVG	2
MK	1
Macular Hole	4
Hyphema	1
FVP	2
Intraop complications, of IOL dropping into vitreous, suprachoroidal hemorrhage and subretinal hemorrhage	1
No. and % of patients needed second surgery	72 (21.2)
Reasons of second VR-surgery (No. and %)	
Recurrent detachment	36 (50.0)
Complications	12 (16.7)
PVR	20 (27.7)
VH	11 (15.3)
Others	15 (20.8)
Macular hole	2

Most of the eyes (288 eyes, 85%) presented with a clear lens at the time of presentation. A total of 51 eyes (15%) had cataract at time of presentation and underwent combined phacovitrectomy during the initial surgery. Additionally, 187 eyes (55.2%) developed cataracts after vitrectomy, requiring cataract surgery.

304 eyes (89.6%) received preoperative bevacizumab injection, and we categorized the timing of this injection into two groups: 3 days or less and more than 3 days. There was no statistically significant difference in the rate of intraoperative hemorrhage between the two groups.

Anatomical success after a single surgery was achieved in 248 eyes (73.2%). Overall anatomical success was achieved in 309 eyes (91.2%). The average number of surgeries required to achieve overall anatomical success was 2.04 surgeries (2–3 surgeries).

Visual success, defined as an improvement in visual acuity of more than two lines on the Snellen chart. Visual success was achieved in 187 eyes (55.2%). Two hundred eyes (69.2%) experienced vision improvement, while 89 eyes (30.7%) had vision deterioration. Patients with vision deterioration were significantly younger than those with vision improvement. The age of presentation in patients with vision deterioration was 28.3 ± 5.9 years, while in patients with vision improvement, it was 30.1 ± 6.8 years (P value = 0.032). Patients with vision deterioation also experienced higher rates of certain complications: 26 eyes (78.8% of all recurrent detachment cases) had recurrent detachment, 19 eyes (82.6% of all PVR cases) had PVR, and 6 eyes (85.7% of all failure of reattachment cases) experienced failure of reattachment.

Table 7 compares various parameters related to both anatomical and visual success. The stage of TRD showed clinically significant results regarding visual success but not anatomical success rate. Simple TRD and complex TRD had better visual success rates compared to combined TRRD (Table 7A).

**Table 7:** Outcomes and factors associated with primary and secondary success

	Primary anatomical uccess	*P* value	Visual success	*P* value
PreOP LogMAR, Mean ± SD	1.5 ± 0.7	0.269	1.7 ± 0.7	< 0.001*
Duration of TRD in months, Mean ± SD	4.1 ± 3.6	0.716	3.9 ± 3.6	0.707
**PreOp evaluation**				
Macula status				
on	59 (83.1)	0.431	49 (71.6)	0.407
off	142 (75.5)		100 (61.0)	
macula on (with foveoschesis)	13 (86.7)		11 (73.3)	
other	34 (73.9)		28 (65.1)	
Stages				
stage 1	3 (75.0)	0.373	3 (75.0)	0.007*
stage 2	9 (100)		7 (87.5)	
stage 3 A	18 (81.8)		14 (66.7)	
stage 3B	34 (77.3)		27 (64.3)	
stage 4	50 (72.5)		40 (65.6)	
stage 5	18 (94.7)		11 (61.1)	
Acute CTRRD	14 (66.7)		7 (38.9)	
chronic CTRRD	51 (78.5)		27 (49.1)	
other	50 (75.8)		50 (82.0)	
Hyaloid status				
Attached	24 (85.7)	0.468	19 (76.0)	0.378
Detached	70 (79.5)		54 (66.7)	
**Intraoperative statistics**				
Intra OP technique				
Bimanual	198 (76.4)	0.203	145 (63.0)	0.100
Unimanual	35 (85.4)		32 (76.2)	
Iatrogenic break				
Yes	88 (73.3)	0.243	59 (57.8)	0.057
No	144 (79.1)		117 (69.2)	
Tamponade				
Yes	182 (76.5)	0.452	128 (59.3)	0.001*
No	66 (80.5)		59 (80.8)	
SO 1300 CS	88 (72.1)	0.452	56 (52.3)	0.001*
SO 5000 CS	39 (79.6)		24 (52.2)	
C3F8	24 (75.0)		25 (86.2)	
SF6	18 (90.0)		18 (81.8)	
other	11 (84.6)		5 (45.5)	
Heavy so	2 (100)		0 (0.0)	
OCT				
ERM	117 (77.0)	0.793	92 (68.7)	0.626
DME	24 (77.4)	0.916	23 (76.7)	0.363
SRF	23 (74.2)	0.600	12 (44.4)	0.003*
ELM status				
Intact	94 (83.9)	0.010*	89 (82.4)	< 0.001*
Attenuated	74 (81.3)		62 (76.5)	
Lost	61 (67.0)		31 (42.5)	
EZ status				
Intact	79 (89.8)	0.005*	70 (84.3)	< 0.001*
Attenuated	61 (74.4)		64 (82.1)	
Lost	89 (71.8)		49 (48.0)	

SD: standard deviation, CTRRD: combined tractional rhegmatogenous retinal detachment, SO: silicone oil, C3F8: perfluoropropane, SF6: sulfur hexafluoride, ERM: epiretinal membrane, DME: diabetic macular edema, SRF: subretinal fluid, ELM: external limiting membrane, EZ: ellipsoid zone

**Table 7a:** Outcomes and factors associated with visual success

Stages	Visual success	*P* value
Simple TRD (Stage 1, 2, 3a)	24 (72.7)	0.374
Complex TRD (Stage 3b + 4 + 5)	78 (64.5)	
Complex TRD	78 (64.5)	0.018*
CTRRD (acute + chronic)	35 (47.3)	
Tamponade		
Gas (C3F8 + SF6)	43 (84.3)	< 0.001*
SO (SO 1300 + SO 5000 + Heavy SO)	80 (51.9)	
C3F8	25 (86.2)	0.713
SF6	18 (81.8)	

TRD: tractional retinal detachment, CTRRD: combined tractional rhegmatogenous retinal detachment, C3F8: perfluoropropane, SF6: sulfur hexafluoride, SO: silicone oil

Eyes that did not receive intraoperative tamponade had a better visual success rate than those that did. Gas tamponade yielded better results compared to silicone oil (SO) regarding visual success (Table 7A). Comparing eyes that received tamponade in terms of visual success, 38 eyes (66.7%) that received gas and 70 eyes (38.7%) that received silicone oil (SO) had a cataract at the time of evaluation at the 1-year postoperative visit. While 8 eyes (14%) that received gas and 84 (46.4%) that received SO were pseudophakic at that time. Postoperative OCT evaluation at 1 year is presented in Table 8.

**Table 8:** Postoperative OCT characteristics at 1-year follow- up

Pathology	No. of eyes
ERM	161 (47.5)
ELM	
Intact	124 (36.6)
Attenuated	93 (27.4)
Lost	93 (27.4)
EZ status	
Intact	96 (28.3)
Attenuated	88 (26.0)
Lost	127 (37.5)
DME	33 (9.7)
SRF	34 (10.0)

ERM: epiretinal membrane, ELM: external limiting membrane, EZ: ellipsoid zone

DME: diabetic macular edema, SRF: subretinal fluid

The multivariate analysis revealed significant predictors for visual success (Table 9). Preoperative LogMAR visual acuity was a significant predictor, with a relative risk (RR) of 9.88 (95% confidence interval [CI]: 4.68–20.83, *p* < 0.001), indicating poorer visual outcomes with higher preoperative LogMAR values. Regarding the stage of TRD, complex TRD was associated with a reduced likelihood of a positive outcome, with an RR of 0.22 (95% CI: 0.05–0.93, *p* = 0.039). Combined TRRD were similarly linked to poorer outcomes, with an RR of 0.08 (95% CI: 0.01–0.5, *p* = 0.007). An intact ellipsoid zone (EZ) status was a strong positive predictor, with an RR of 20.4 (95% CI: 3.8–109.41, *p* < 0.001). Additionally, the absence of subretinal fluid on postoperative OCT was associated with positive outcomes, with an RR of 3.49 (95% CI: 1.12–10.87, *p* = 0.031).

**Table 9:** Multivariate analysis – independent predictors of visual success

Predictor	RR	95% CI	*P* value
Preoperative LogMAR VA	9.88	4.68–20.83	< 0.001*
Complex TRD (vs. Simple)	0.22	0.05–0.93	0.039*
Combined TRRD (vs. Simple)	0.08	0.01–0.50	0.007*
Intact EZ status	20.40	3.80–109.41	< 0.001*
Absence of subretinal fluid	3.49	1.12–10.87	0.031*

RR: relative risk, CI: confidence interval, VA: visual acuity, TRD: tractional retinal detachment, TRRD: combined tractional rhegmatogenous retinal detachment, EZ: ellipsoid zone. *Statistically significant (*p* < 0.05)

## Discussion

This study evaluated 339 eyes from 255 patients with T1DM who underwent PPV for TRD and combined TRRD. The key findings are that simple and complex TRD demonstrated better visual acuity improvement compared to combined TRRD. Eyes without tamponade achieved superior visual results. Gas tamponade yielded better visual outcomes than silicone oil tamponade. The integrity of the external limiting membrane (ELM) and the ellipsoid zone (EZ) after surgery was strongly associated with visual acuity improvement.

A critical contribution of this study is the introduction of a proposed TRD staging system designed to address limitations of existing classifications in the context of contemporary MIVS PPV. Several classification systems have been proposed for TRD, with Kroll et al.’s system being among the most cited [8]. Kroll’s classification of proliferative diabetic vitreoretinopathy (PDVR) described three stages: stage A (proliferative changes with attached retina), stage B (extramacular TRD), and stage C (TRD involving the macula). While this system demonstrated prognostic value, showing significantly more improvement in visual acuity in stage A compared to stages B and C, it has notable limitations for modern surgical practice. Specifically, it places excessive focus on surgical anatomy without adequately addressing hyaloid status, does not distinguish between the chronicity of detachment, and does not account for the presence of rhegmatogenous components that fundamentally alter the surgical approach and prognosis.

Other classification approaches have also been described. Zarbin and colleagues introduced a “complexity score” based on the number of quadrants of fibrovascular proliferation, the location of proliferation relative to the equator, the type of retinal detachment (tractional vs. traction-rhegmatogenous), and the presence or absence of posterior vitreous detachment [10, 11]. This system was designed primarily to compare cases of similar surgical difficulty and was applied in studies evaluating silicone oil tamponade and viscodissection techniques in severe proliferative diabetic retinopathy. While the complexity score provides a useful quantitative measure of surgical difficulty, it does not incorporate the chronicity of detachment or the status of outer retinal structures, and it was not specifically designed as a prognostic staging system. Hsu et al. proposed a grading system for combined tractional and rhegmatogenous retinal detachment in proliferative diabetic retinopathy, classifying the severity of vitreoretinal adhesion into four grades based on the extent and location of fibrovascular proliferation relative to the equator [12]. Their study demonstrated that higher grades of vitreoretinal adhesion, broader extent of retinal detachment, and intraoperative retinectomy were significant predictors of poor visual outcomes. However, this system was focused specifically on combined tractional-rhegmatogenous detachments and does not encompass the full spectrum of TRD presentations, including purely tractional cases with varying degrees of hyaloid attachment. In comparison, our proposed staging system aims to provide a more comprehensive classification that encompasses the entire spectrum of TRD, from simple focal traction to complex cases with complete hyaloid attachment and combined rhegmatogenous components, thereby offering broader applicability for contemporary surgical decision-making in T1DM.

Our proposed staging system addresses these gaps by incorporating three key dimensions: the extent and location of tractional membranes (focal vs. broad, macular involvement), the status of posterior hyaloid attachment (partial vs. complete posterior vitreous detachment), and the presence and chronicity of retinal breaks (distinguishing acute from chronic combined TRRD). This system allows differentiation between simple TRD (stages 1–3a) and complex TRD (stages 3b–5), as well as between acute and chronic combined TRRD, providing a more granular framework for prognostic assessment and surgical planning. It is important to emphasize that this staging system is proposed as a hypothesis-generating framework. It has been developed based on retrospective surgical observations and requires validation through prospective multicenter studies with inter-observer agreement analysis before it can be adopted for clinical decision-making.

We found that simple TRD, complex TRD, and combined TRRD had similar anatomical success rates after a single surgery. However, simple and complex TRD achieved better visual acuity improvement, with combined TRRD demonstrating significantly lower visual success (47.3% vs. 64.5% for complex TRD, *p* = 0.018).

In our study, the mean time between presentation and surgery ranged from the same day to 1121 days (153.4 ± 221.6 days). This large variation can be attributed to patients who initially had extramacular TRD that did not require surgical intervention but progressed to involve the macula during regular follow-ups. Some patients also developed VH, leading to a significant decrease in vision and necessitating surgical intervention.

Unimanual or bimanual techniques can be used in vitrectomy with membrane peeling. Bimanual techniques achieved similar anatomical and visual outcomes to unimanual but offered advantages in surgical time, membrane removal time, and fewer iatrogenic breaks [13]. Complication rates were similar, though some studies noted higher hypotony in bimanual groups [14]. Our study did not find a significant difference in success rates or rate of complications between the two techniques.

Various ocular tamponades, including silicone oil, gas, or air, can be utilized during vitrectomy. Rush et al. compared silicone oil to gas tamponade in diabetic TRD and found that perfluoropropane gas resulted in significantly better visual outcomes at 6 months [15]. This may be related to thinner retinal layers observed on OCT, although this association was not statistically significant in their study. Christensen et al. found a statistically significant correlation between poor visual outcomes and thinner inner retinal layers in the silicone oil group [16]. 

Our study found higher visual success rates associated with gas tamponades (C3F8 and SF6) compared to silicone oil (84.3% vs. 51.9%, respectively) at 1 year postoperatively. However, eyes that did not receive any tamponade achieved better visual outcomes: 80.8% of these eyes had visual success compared to 59.3% of eyes that received tamponade (*p* = 0.001). It is critical to note that these tamponade-related findings represent associations rather than causal relationships. The poorer visual outcomes observed in the silicone oil group are likely confounded by indication bias, as silicone oil was preferentially used in more severe and complicated cases. Indeed, silicone oil was the most commonly used tamponade in both complex TRD and combined TRRD, accounting for 65 eyes (46.1%) and 88 eyes (80%), respectively. Future randomized or protocol-based studies comparing tamponade agents in stratified TRD stages are needed to establish any causal relationship.

Tao et al. conducted a study assessing the outcomes of vitrectomy in TRD without tamponade, concluding that 94% of patients achieved successful retinal reattachment with improvement in visual acuity in 75% of cases [17]. Storey et al. found anatomical success in 87.6% of diabetic TRD surgeries, with silicone oil tamponade yielding inferior outcomes [18]. They hypothesized silicone oil tamponade eyes had more complex TRD, with half having CTRRD and 75% macula-involved. Another study found that factors associated with a poor prognosis after vitrectomy for diabetic retinopathy included macular detachment, the use of long-acting intraocular tamponade and poor preoperative vision in both the operated eye and the contralateral eye [19]. The authors suggested the poorer outcomes associated with long-acting tamponade are due to the complexity of dissection during surgery rather than a direct effect of the tamponade itself. Our results are largely concordant with these findings, with our overall anatomical success rate of 91.2% and single-surgery success of 73.2%. Notably, our cohort is exclusively composed of T1DM patients with a mean age of 30.0 years, which distinguishes it from mixed or predominantly T2DM cohorts studied previously. This younger demographic may partially account for differences in tamponade response and outcomes, as T1DM patients tend to have more firmly attached posterior hyaloid, more aggressive fibrovascular proliferation, and longer disease duration.

The multivariate analysis identified several independent predictors of visual success that merit discussion. Preoperative LogMAR visual acuity was the strongest predictor (RR 9.88), confirming that better baseline vision is associated with significantly improved postoperative outcomes. This finding underscores the importance of timely surgical intervention before severe vision loss occurs. The stage of TRD was also a significant independent predictor, with complex TRD (RR 0.22) and combined TRRD (RR 0.08) associated with markedly reduced likelihood of visual success compared to simple TRD. Intact EZ status emerged as the strongest structural predictor (RR 20.4), indicating that photoreceptor integrity is a key determinant of functional recovery. The absence of subretinal fluid on postoperative OCT (RR 3.49) further reinforced the importance of structural restoration in achieving visual success. Previous studies described multiple factors affecting the outcomes of vitrectomy in diabetic TRD. Better visual outcomes were associated with factors such as preoperative visual acuity of 5/200 or better, absence of iris neovascularization, clear lens, and prior pan-retinal photocoagulation (PRP). Conversely, poorer visual outcomes were associated with intraoperative lens removal and the creation of iatrogenic retinal breaks [20, 21].

The integrity of the external limiting membrane (ELM) and the ellipsoid zone (EZ) after surgery was strongly associated with visual acuity improvement. We found that 82.4% of patients with an intact ELM and 84.3% with an intact EZ experienced improved visual acuity of more than two lines (visual success), compared to only 42.5% and 48% of patients with disrupted ELM and EZ, respectively. No other OCT findings were associated with significant results. Dooley et al. found postoperative disruption of the photoreceptor inner and outer segment (IS/OS) junction and the ELM was associated with poorer visual outcomes [9]. 

The physiological basis for these findings relates to the role of the ELM and EZ as indicators of photoreceptor and outer retinal health. Prolonged retinal detachment, ischemic damage, and chronic traction can cause irreversible disruption of these structures, leading to permanent photoreceptor loss. Our large T1DM series supports and extends prior OCT studies, establishing ELM/EZ as relevant structural biomarkers in surgeries for advanced proliferative diabetic retinopathy. These findings suggest that postoperative OCT is not only valuable for structural assessment but may also serve as a crucial prognostic tool in routine clinical practice and future research endeavors, helping clinicians counsel patients regarding expected visual recovery.

In our study, the timing of preoperative anti-VEGF injection did not significantly impact the rate of intraoperative hemorrhage. This finding aligns with the results of Castillo et al., who reported no significant difference in intraoperative complications between patients receiving anti-VEGF injections 1–3 days versus 5–10 days before vitrectomy [22]. 

Few studies have compared the outcomes of PPV in patients with T1DM and T2DM [7, 23]. Kumar et al. found lower final retinal reattachment in T1DM (88.1%) vs. T2DM (96.6%), with significant BCVA improvement in T2DM. Kazmierczak et al. found anatomical success in 88.9% T1DM and 95.5% T2DM, with more significant BCVA improvement in T2DM. The reasons for these differences are likely multifactorial, including longer duration of diabetes, worse glycemic control, associated comorbidities, and a higher incidence of macular tractional retinal detachment in patients with T1DM.

Huang et al. found worse outcomes in patients under 40, with higher recurrent detachment and poorer final visual acuity. The authors suggested that poorer results could be due to several factors including severe retinopathy, fibrovascular proliferation, TRD extent, longer diabetes duration, severe ischemia, surgery time, inflammation, and IOP fluctuation [24]. One of the challenges in T1DM is the firmly attached posterior hyaloid, which can lead to poorer surgical outcomes in those patients [25]. 

### Future directions

Several avenues for future research emerge from this study. First, prospective multicenter validation of the proposed TRD staging system is essential, including formal inter-observer agreement studies to ensure consistency and reproducibility. Second, randomized or protocol-based studies comparing gas and silicone oil tamponade in cohorts stratified by TRD stage and macular status are needed to establish evidence-based tamponade selection criteria. Third, serial OCT studies assessing temporal changes in ELM/EZ integrity and subretinal fluid resolution would provide valuable insight into the dynamics of functional recovery and optimal timing for prognostic assessment. Fourth, integration of systemic parameters such as HbA1c levels and nephropathy severity into prognostic models would allow for more comprehensive outcome prediction. These future investigations would build upon the present findings and advance evidence-based management of TRD in T1DM.

This study’s strength lies in its large sample size, providing extensive data on PPV outcomes for tractional retinal detachment in T1DM. Another strength is the introduction of a proposed staging system for TRD, which offers a more standardized approach to classifying the severity of the condition. The study also provides one of the largest assessments of outer retinal biomarkers (ELM/EZ) in a purely T1DM TRD cohort.

However, several important limitations must be acknowledged. First, the retrospective single-center design limits external validity and introduces potential selection and recall bias. Second, there is significant indication bias in tamponade selection, as silicone oil was preferentially used in more complex cases, which may overestimate its negative impact on visual outcomes; the direction of this bias favors worse outcomes in the silicone oil group. Third, the absence of systemic data, including HbA1c levels, blood pressure control, and nephropathy severity, limits the comprehensiveness of the prognostic model and may result in residual confounding. Fourth, imaging data were not comprehensive for all patients, with 69 eyes (20.7%) lacking staging data due to unavailable fundus photographs. While color fundus photographs were used to illustrate the proposed staging system, the addition of optical coherence tomography (OCT) and B-scan ultrasonography imaging would further enhance the characterization and understanding of each stage; future studies incorporating multimodal imaging are warranted to provide a more complete anatomical depiction of the staging system. Fifth, the proposed staging system lacks inter-observer agreement data and was assessed by multiple surgeons without a standardized grading protocol, which may introduce classification variability. Sixth, the inclusion of bilateral eyes from the same patient (84 patients, 168 eyes) introduces potential clustering effects that were not addressed by mixed-effects modeling. Seventh, OCT graders were not blinded to clinical data, which may introduce assessment bias. Eighth, surgical techniques and instrumentation may have evolved over the 8-year study period, potentially affecting the consistency of results. According to the GRADE framework, the quality of evidence from this retrospective cohort study is considered “low.” Future prospective studies are needed to confirm these findings and explore other potential influences on PPV success in T1DM.

## Conclusion

With current techniques, PPV for TRD and combined TRRD in T1DM achieves excellent overall anatomical outcomes, with an overall reattachment rate of 91.2%. The proposed TRD staging system demonstrated prognostic relevance, with combined TRRD carrying a significantly worse visual prognosis compared to simple and complex TRD. Preoperative visual acuity, TRD stage, outer retinal structural integrity (ELM/EZ) on postoperative OCT, and tamponade type were significant determinants of functional outcomes. The association of silicone oil tamponade with poorer visual outcomes likely reflects indication bias inherent in the retrospective design rather than a direct detrimental effect. The proposed staging system is hypothesis-generating and requires prospective multicenter validation with inter-observer agreement studies.

## Data Availability

the data generated during the current study are stored in the hospital electronic medical record system and are not publicly available due to patient confidentiality and institutional data protection policies. The data are available from the corresponding author upon reasonable request and with permission from the institution.

## Abbreviations

BCVA: Best-corrected visual acuity
C3F8: Perfluoropropane
DR: Diabetic retinopathy
DRVS: Diabetic Retinopathy Vitrectomy Study
ELM: External limiting membrane
ETDRS: Early Treatment Diabetic Retinopathy Study
EZ: Ellipsoid zone
IS/OS: Photoreceptor inner and outer segment
MIVS: Minimally invasive vitreoretinal surgery
OCT: Optical coherence tomography
PDVR: Proliferative diabetic vitreoretinopathy
PPV: Pars plana vitrectomy
PRP: Panretinal photocoagulation
SF6: Sulfur hexafluoride
SO: Silicone oil
T1DM: Type 1 diabetes mellitus
T2DM: Type 2 diabetes mellitus
TRD: Tractional retinal detachment
TRRD: Tractional rhegmatogenous retinal detachment
VEGF: Vascular endothelial growth factor
VH: Vitreous hemorrhage
VTDR: Vision-threatening diabetic retinopathy
